# The Association between Liver Enzymes and Mortality Stratified by Non-Alcoholic Fatty Liver Disease: An Analysis of NHANES III

**DOI:** 10.3390/nu15133063

**Published:** 2023-07-07

**Authors:** Saskia Rita Grob, Flurina Suter, Verena Katzke, Sabine Rohrmann

**Affiliations:** 1 Medical Faculty, University of Zurich, Pestalozzistrasse 3, CH-8032 Zurich, Switzerland; saskiarita.grob@uzh.ch; 2 Division of Chronic Disease Epidemiology, Epidemiology, Biostatistics and Prevention Institute (EBPI), University of Zurich, Hirschengraben 84, CH-8001 Zurich, Switzerland; flurina.suter2@uzh.ch; 3 Division of Cancer Epidemiology, German Cancer Research Center (DKFZ), Im Neuenheimer Feld 280, DE-69120 Heidelberg, Germany; v.katzke@dkfz-heidelberg.de

**Keywords:** mortality, liver enzymes, De Ritis ratio, non-alcoholic fatty liver disease, hepatic steatosis

## Abstract

Associations between liver enzymes or De Ritis ratio (DRR; aspartate aminotransferase (AST)/alanine aminotransferase (ALT)) and mortality stratified by non-alcoholic fatty liver disease (NAFLD), which have rarely been analyzed in previous studies, were investigated using the National Health and Nutrition Examination Survey (NHANES) III (1988–1994). Participants without risk factors for liver diseases other than NAFLD were linked with National Death Index records through 2019 (*n* = 11,385) and divided into two cohorts with or without NAFLD, based on ultrasound examination. Liver enzyme concentrations were categorized into sex-specific deciles and subsequently grouped (AST and ALT: 1–3, 4–9, 10; gamma glutamyltransferase (GGT): 1–8, 9–10). DRR was categorized into tertiles. Cox proportional hazards regression models adjusted for confounders were fitted to estimate associations with mortality. Compared with low levels, high GGT and DRR in participants with and without NAFLD had significantly higher hazard ratios for all-cause mortality. Compared with intermediate concentrations, low ALT showed higher all-cause mortality in participants with and without NAFLD, whereas low AST had higher HR in participants without NAFLD and high AST in those with NAFLD. Mortality was associated with liver enzymes or DRR in participants both with and without NAFLD, indicating that the relationship is not mediated solely by hepatocellular damage.

## 1. Introduction

Liver enzymes are not only markers of hepatocellular or cholestatic damage [1] but have also been shown to be associated with mortality [2,3,4,5,6]. Elevated transaminases and gamma glutamyltransferase (GGT) were significantly associated with mortality from liver disease [4,7]. However, liver enzymes have also been shown to be associated with all-cause, cardiovascular disease, and cancer mortality [2,3,5,7]. Thereby, the relationship between alanine aminotransferase (ALT) or aspartate aminotransferase (AST) and mortality was described as U- or J-shaped [2,5], and low transaminases appear to be more likely to be related to mortality than high transaminases compared with intermediate concentrations [3,4,5,6]. The De Ritis ratio (DRR), defined as the ratio of AST over ALT [8], was likewise associated with all-cause, cardiovascular disease, and cancer mortality [3]. Many potential mechanisms have been discussed to explain the association between liver enzymes and mortality, such as oxidative stress, inflammation, hepatic aging, malnutrition, frailty, and lack of specificity of liver enzymes for hepatocellular damage [1,9,10,11,12]. Nevertheless, Haring et al. showed a stronger association between GGT and mortality in men with hepatic hyperechogenicity than in those without [13]. With a prevalence of 25%, non-alcoholic fatty liver disease (NAFLD) is the leading cause of chronic liver disease worldwide [14] as well as the leading cause of elevated liver enzyme concentrations in the United States [15]. The progressive form of NAFLD, non-alcoholic steatohepatitis (NASH), is expected to soon become the most common indication for liver transplantation [16]. However, NAFLD is a complex multisystem disease that is associated with metabolic syndrome (MetS) and not only leads to increased liver morbidity and mortality but also puts patients with NAFLD at an increased risk of developing cardiovascular diseases and extrahepatic malignancies, which are the leading causes of death in this patient population [14]. It has previously been discussed whether the relationship between liver enzymes and mortality is due to MetS, which is associated with NAFLD and many non-liver diseases [17,18]. Nevertheless, in previous studies, NAFLD seemed to be only inconsistently associated with mortality [4,19,20]. Furthermore, liver enzymes correlated poorly with NAFLD severity [21,22,23,24]. Liver enzyme concentrations can be in the normal range in over 50% of patients with NAFLD [21]. Given the importance of NAFLD and its potential role in the association between liver enzymes and mortality, this study investigates whether there was a difference in the relationship between mortality and liver enzymes when stratifying the participants by NAFLD diagnosis. Despite the high burden of disease, hardly any study that has examined the association between mortality and liver enzymes has screened its participants for NAFLD and subsequently excluded NAFLD or distinguished between participants with and without NAFLD. We used data from the well-described, cross-sectional, nationally representative National Health and Nutrition Examination Survey (NHANES) III study with up to 31 years of follow-up.

## 2. Materials and Methods

### 2.1. Subjects and Study Design

NHANES III is a nationally representative survey of the civilian, non-institutionalized United States population consisting of interviews, physical examinations, and laboratory testing. It was conducted from 1988 to 1994 by the National Center for Health Statistics (NCHS) of the Centers for Disease Control and Prevention (CDC). Data were collected from a complex multistage, stratified, clustered probability sample with oversampling of persons aged 60 years or older, non-Hispanic blacks, Mexican Americans, and children between two months and five years of age [25]. NHANES III was approved by the institutional review board of the CDC, and all participants provided written informed consent. NHANES III data are freely available from the U.S. Centers for Disease Control and Prevention (https://wwwn.cdc.gov/nchs/nhanes/nhanes3/datafiles.aspx (accessed on 17 June 2022)).

Of the 20,050 adults who completed the interview, 18,825 were 20 years or older. To exclude participants with risk factors for liver disease other than NAFLD, we excluded men drinking >2 drinks/d and women drinking >1 drink/d (*n* = 596) [26]; pregnant women (*n* = 228); participants with a serum transferrin saturation >50% (*n* = 463); positive or borderline positive or missing serum hepatitis B surface antigen or serum hepatitis C antibody (*n* = 3540); participants who were missing data on transferrin saturation (*n* = 68), alcohol consumption (*n* = 35), mortality follow-up (*n* = 10), and abdominal ultrasonography; and participants with abdominal ultrasonography that was ungradable for hepatic steatosis (*n* = 2233). In addition, we excluded participants with missing values for any covariate included in the multivariate-adjusted Cox analyses (age, sex, race/ethnicity, education, alcohol consumption, cigarette smoking, leisure-time physical activity (LTPA), body mass index (BMI), diabetes, hypertension, high density lipoprotein (HDL), total cholesterol, triglycerides; *n* = 196). For analyses of liver enzymes and DRR, participants with missing data on ALT (*n* = 71), AST (*n* = 71), or GGT (*n* = 2577) were excluded. After applying these exclusion criteria, our study samples for AST, ALT, and DRR consisted of 11,385 total participants and 8879 for GGT. Two participants with missing cause of death were excluded for the analyses of cause-specific mortality.

### 2.2. Measurement of Hepatic Steatosis and Liver Enzymes

We defined NAFLD as sonographically graded moderate to severe hepatic steatosis in the absence of other causes for fatty liver disease. The hepatic steatosis ultrasound examination was conducted between 2009 and 2010 by reviewing archived gallbladder ultrasound-examination videotapes of adults aged 20 to 74 years, which were originally obtained during the mobile examination center (MEC) examination in NHANES III. Hepatic steatosis was evaluated based on the presence or absence of five criteria: parenchymal brightness, liver to kidney contrast, deep beam attenuation, bright vessel walls, and gallbladder wall definition. Hepatic steatosis was graded as normal, intermediate (mild), moderate, or severe and dichotomized as absent (normal-mild) or present (moderate-severe). The full protocol can be found elsewhere [27].

After collection, the serum samples were shipped to the testing laboratories at −20 °C. Analyses of serum ALT, AST, and GGT were performed using Hitachi Model 737 multichannel analyzer (Boehringer Mannheim Diagnostics, Indianapolis, IN) [28]. Due to different activities of liver enzymes in men and women, we categorized the enzymes as sex-specific deciles. The relationship of ALT and AST with mortality was previously described to be U- or J-shaped [2,5]. Therefore, we combined the sex-specific deciles into three categories, i.e., 1–3, 4–9, and 10. The deciles 4–9 served as the reference group. The AST cut-points were 18 and 34 U/L for men and 16 and 27 U/L for women. The ALT cut-points were 13 and 36 U/L for men and 9 and 24 U/L for women. GGT was dichotomized into deciles 1–8, serving as the reference group, and deciles 9–10, because a linear relationship between GGT and mortality has been described [2]. The GGT cut-points were 46 U/L for men and 31 U/L for women. DRR was calculated as the ratio of AST over ALT [8] and divided into tertiles with cut-points of 1.2 and 1.64. The first tertile served as the reference group.

### 2.3. Variables of Interest

We included the following variables in our analyses: age (years), sex (male, female), race/ethnicity (non-Hispanic white, non-Hispanic black, Mexican American, other), education (years; <12, 12, >12), alcohol consumption (0 drinks/day, men ≤2 or women ≤1 drinks/day), cigarette smoking (never, former, current), LTPA (no, irregular, regular), BMI (weight [kg]/height [m^2^]), diabetes, physician-diagnosed hypertension, frailty, concentrations of HDL (mg/dL; <35, ≥35), total cholesterol (mg/dL; <200, 200–239, ≥240), triglycerides (mg/dL; <250, 250–500, >500) [28], albumin (g/dL), total bilirubin (mg/dL), platelets (G/L), C-reactive protein (CRP) (mg/dL; ≤0.3, >0.3) [28], and estimated glomerular filtration rate (eGFR) (ml/min/1.73 m^2^; <60, ≥60). However, in the multivariate-adjusted Cox proportional hazard regression model, no adjustment was made for bilirubin, platelets, albumin, CRP, or eGFR because we intentionally prioritized their role as potential mediators between NAFLD and mortality rather than as potential confounders. Measurement protocols for variables assessed in NHANES III are described elsewhere [25].

LTPA was categorized in moderate and vigorous activity by the intensity and frequency of an activity (details are described elsewhere [29]). It was further categorized as “no LTPA” (no vigorous or moderate physical activity), “irregular LTPA” (≤4 times/week moderate activity or ≤2 times/week vigorous activity), and “regular LTPA” (>4 times/week moderate activity or >2 times/week vigorous activity) [30]. Diabetes was defined as physician-diagnosed diabetes or concentration of hemoglobin A1c (HbA1c) ≥6.5% [31]. The eGFR was calculated using the 2021 Chronic Kidney Disease Epidemiology Collaboration (CKD-EPI) equation [32,33]. For assessing frailty, a version of the frailty score originally developed by Fried et al. and adapted to the NHANES III data by Wilhelm-Leen et al. was used [34,35]. Participants with valid information on at least three frailty criteria were included and classified as frail if they met at least three of the five criteria [35,36].

### 2.4. Mortality Follow-Up

NHANES III participants were linked with National Death Index (NDI) records through 31 December 2019. Cause of death was categorized into 9 leading causes of death according to the International Classification of Diseases, 9th (ICD-9) and 10th (ICD-10) Revisions. Deaths originally coded under ICD-9 codes were recoded with ICD-10 codes. Heart disease cause of death was defined by the ICD-10 codes I00-I09, I11, I13, I20-I51, and cancer cause of death was defined by the ICD-10 codes C00-C97 [37,38].

### 2.5. Statistical Analysis

To calculate cumulative mortality, we conducted a Kaplan-Meier survival analysis. Cox proportional hazard regression analysis was used to compare enzyme deciles or DRR tertiles while adjusting for potential risk factors. Confounders were selected a priori based on known relationships with liver enzymes and outcomes. The following covariates were included in the multivariate-adjusted Cox proportional hazard regression model: age, sex, race/ethnicity, education, alcohol consumption, cigarette smoking, LTPA, BMI, diabetes, hypertension, HDL, total cholesterol, and triglycerides. The alcohol consumption, triglycerides, HDL, and total cholesterol covariates were categorized as groups due to an observed right-skewed distribution. We reran the Cox proportional hazard regression analyses excluding participants taking certain medications that may cause hepatic steatosis (amiodarone, nucleoside analog reverse transcriptase inhibitors (NRTI; didanosine, zidovudine, zalcitabine), tamoxifen, methotrexate, fluorouracil, glucocorticoids (beclomethasone, betamethasone, cortisone, hydrocortisone, dexamethasone, methylprednisolone, prednisolone, triamcinolone); *n* = 10 in analysis samples for AST, ALT, and DRR, *n* = 8 in analysis sample for GGT), additionally adjusting the multivariate-adjusted Cox model for frailty once. The model, which was additionally adjusted for frailty, excluded participants with missing values for frailty (*n* = 3986 in analysis samples for AST, ALT, and DRR, *n* = 2132 in analysis sample for GGT). Time at risk was defined as time from the date of the interview to the date of death or to 31 December 2019. For analyses of cause-specific mortality, the participants who died from other causes were censored at the date of death. The proportional hazard assumption was verified using scaled Schoenfeld residual plots. Accounting for the complex survey design of NHANES III, sample weights were used for all analyses. Variance calculations were conducted using Taylor series linearization. A *p*-value of <0.05 was considered to indicate statistical significance. All statistical analyses were performed using R software version 4.2.0 (R Core Team (2022). (R: A language and environment for statistical computing. R Foundation for Statistical Computing, Vienna, Austria. URL https://www.R-project.org/).

## 3. Results

Among the 11,385 participants in the analysis sample for AST, ALT, and DRR, 2643 (23.2%) were sonographically diagnosed with moderate to severe hepatic steatosis, while in the analysis sample for GGT, 2075 (23.4%) of the 8879 participants were sonographically diagnosed with moderate to severe hepatic steatosis.

In both cohorts, participants with hepatic steatosis appeared to be older, to be more likely to be male, and to have diabetes, hypertension, dyslipidemia, elevated CRP, low eGFR, and higher BMI compared with participants without hepatic steatosis (Table 1). Compared with the respective reference deciles, participants with liver enzyme concentrations in the highest deciles seemed to be more likely to be female, Mexican American, and diabetic, to have dyslipidemia and elevated CRP, to be less likely to be non-Hispanic white, and to have higher BMI, with or without NAFLD (Supplementary Tables S1–S3). Participants with and without hepatic steatosis with GGT levels in the highest deciles seemed to be older, whereas those with ALT activity in the highest decile appeared to be younger compared with their respective reference deciles.

Compared with the first tertile, participants in the third DRR tertile with and without hepatic steatosis seemed to be older, more likely to be female, and non-Hispanic black and to have elevated CRP and low eGFR. They were also less likely to be Mexican American, to be diabetic, and to have dyslipidemia and appeared to have lower BMI (Supplementary Table S4).

Participants in the highest deciles of GGT with and without hepatic steatosis and those with ALT in decile 10 with hepatic steatosis as well as those with DRR in tertile 3 without hepatic steatosis seemed to be more likely to be frail compared with the respective reference deciles or tertile.

The median follow-up time among all participants included in the analysis sample for AST, ALT, and DRR was 27.1 years (interquartile range (IQR) 25.1–28.8 years, range 0.17–31.2 years). In this analysis sample, cumulative mortality from all causes at 31 years of follow-up was 30.4% (2808 deaths) in participants without hepatic steatosis and 44.6% (1192 deaths) in participants with hepatic steatosis. Cause-specific cumulative mortality was 9.3% (785 deaths) from heart disease, 9.4% (693 deaths) from cancer in participants without hepatic steatosis, 13.9% (332 deaths) from heart disease, and 12.2% (260 deaths) from cancer in participants with hepatic steatosis.

Among all participants included in the GGT cohort, the median follow-up time was 26.8 years (IQR 25.3–28.2 years, range 0.17–31.1 years). In the analysis sample for GGT, cumulative mortality from all causes after 31 years of follow-up was 27.2% (2076 deaths) in participants without hepatic steatosis and 39.9% (884 deaths) in those with hepatic steatosis. Cause-specific cumulative mortality was 7.4% (564 deaths) from heart disease, 8.3% (515 deaths) from cancer in participants without hepatic steatosis and 12.0% (247 deaths) from heart disease, and 10.5% (199 deaths) from cancer in participants with hepatic steatosis.

**Figure 1:**
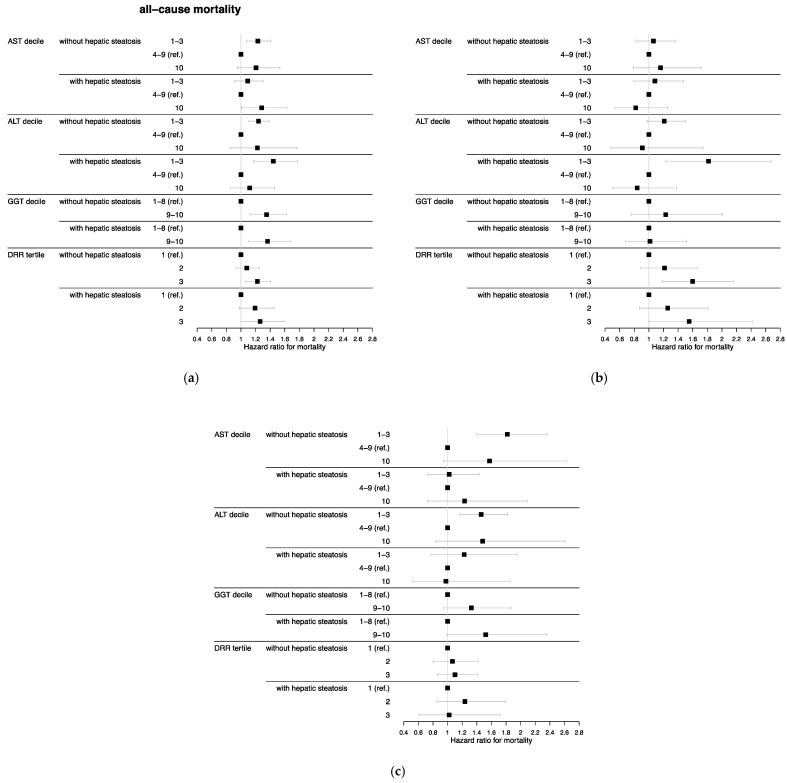
Multivariate-adjusted hazard ratios with 95% confidence intervals for (**a**) all-cause, (**b**) heart disease, and (**c**) cancer mortality by hepatic steatosis and liver enzyme decile or DRR tertile (*n* = 11,385 in analysis samples for AST, ALT, and DRR; *n* = 8879 in analysis sample for GGT) of the National Health and Nutrition Examination Survey III (1988–1994), United States. Abbreviations: AST = aspartate aminotransferase, ALT = alanine aminotransferase, GGT = gamma glutamyltransferase, DRR = De Ritis ratio. AST cut-points were 18 and 34 U/L for men and 16 and 27 U/L for women. ALT cut-points were 13 and 36 U/L for men and 9 and 24 U/L for women. GGT cut-points were 46 U/L for men and 31 U/L for women. DRR cut-points were 1.2 and 1.64. Hazard ratios were estimated using Cox proportional hazard regression analysis and adjusted for age, sex, race/ethnicity, education, alcohol consumption, cigarette smoking, leisure-time physical activity, body mass index, diabetes, hypertension, high-density lipoprotein, total cholesterol, and triglycerides.

After adjusting for age, HR for all-cause mortality was higher among participants in AST and ALT deciles 1–3 both with and without hepatic steatosis compared with the reference deciles 4–9 (Table 2). The relationship stayed significant in multivariate-adjusted analyses except for participants with hepatic steatosis in the lowest AST deciles (AST with NAFLD: hazard ratio (HR) = 1.09, 95% confidence interval (CI) = 0.91–1.31; AST without NAFLD: HR = 1.23, 95% CI = 1.08–1.41; ALT with NAFLD: HR = 1.44, 95% CI = 1.17–1.78; ALT without NAFLD: HR = 1.24, 95% CI = 1.11–1.39) (Figure 1). Furthermore, AST in the highest decile in participants with hepatic steatosis was associated with all-cause mortality compared with the reference categories (HR = 1.28, 95% CI = 1.01–1.63). Both low AST and ALT without hepatic steatosis were associated with cancer mortality compared with deciles 4–9 (AST without NAFLD: HR = 1.82, 95% CI = 1.40–2.36; ALT without NAFLD: HR = 1.46, 95% CI = 1.17–1.82). For heart disease, participants in ALT deciles 1–3 with and without hepatic steatosis as well as low AST without hepatic steatosis were associated with higher risk of mortality compared with the reference deciles after adjusting for age. The risk only remained significant in multivariate-adjusted analysis for low ALT in participants with hepatic steatosis (HR = 1.82, 95% CI = 1.23–2.67).

High GGT in participants with and without hepatic steatosis had higher HR for all-cause mortality compared with deciles 1–8 after adjusting for age (Table 2) and remained significant in multivariate-adjusted analyses (GGT with NAFLD: HR = 1.36, 95% CI = 1.11–1.68; GGT without NAFLD: HR = 1.35, 95% CI = 1.12–1.62) (Figure 1). No significant association was observed between GGT and cancer or heart disease mortality.

After adjusting for age, neither DRR tertile 2 nor tertile 3 were significantly associated with mortality compared with tertile 1 (Table 2). However, after adjusting for multiple factors, participants in DRR tertile 3 with (HR = 1.26, 95% CI = 1.00–1.59) and without hepatic steatosis (HR = 1.22, 95% CI = 1.07–1.41) showed significant associations with all-cause mortality compared with the reference tertile (Figure 1). Furthermore, tertile 3 in participants without hepatic steatosis had a higher association with heart disease mortality compared with tertile 1 (HR = 1.60, 95% CI = 1.18–2.16).

We conducted a sensitivity analysis on the multivariate-adjusted Cox analyses excluding participants who were taking medication that could cause hepatic steatosis. There was little effect on the results (Supplementary Figure S1).

**Figure 2:**
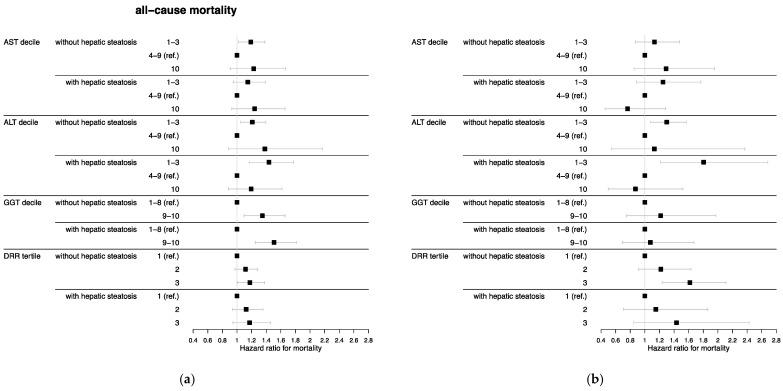

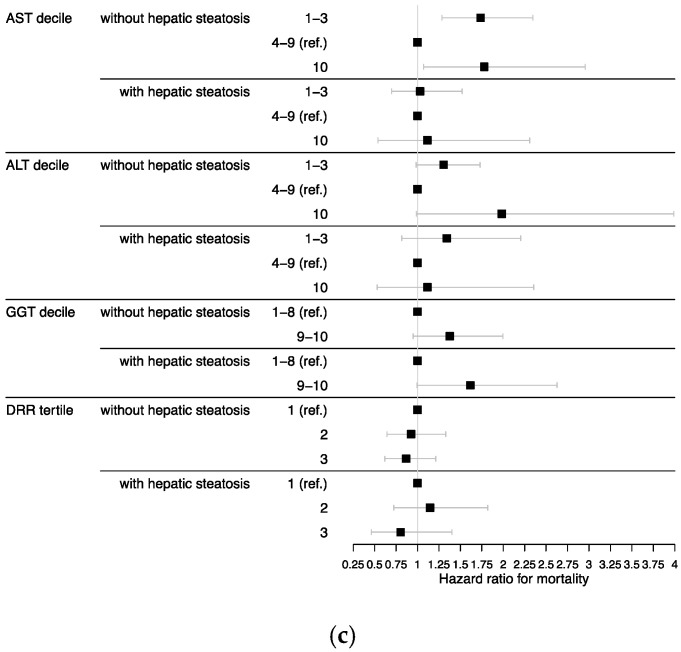
Hazard ratios additionally adjusted for frailty with 95% confidence intervals for (**a**) all-cause, (**b**) heart disease, and (**c**) cancer mortality by hepatic steatosis and liver enzyme decile or DRR tertile (*n* = 7399 in analysis samples for AST, ALT, and DRR; *n* = 6747 in analysis sample for GGT) of the National Health and Nutrition Examination Survey III (1988–1994), United States. Abbreviations: AST = aspartate aminotransferase, ALT = alanine aminotransferase, GGT = gamma glutamyltransferase, DRR = De Ritis ratio. AST cut-points were 18 and 34 U/L for men and 16 and 27 U/L for women. ALT cut-points were 13 and 36 U/L for men and 9 and 24 U/L for women. GGT cut-points were 46 U/L for men and 31 U/L for women. DRR cut-points were 1.2 and 1.64. Hazard ratios were estimated using Cox proportional hazard regression analysis and adjusted for age, sex, race/ethnicity, education, alcohol consumption, cigarette smoking, leisure-time physical activity, body mass index, diabetes, hypertension, high-density lipoprotein, total cholesterol, triglycerides, and frailty.

Moreover, we reran the multivariate-adjusted Cox analyses additionally adjusted for frailty, which resulted in some alterations of significant associations (Figure 2). The relationship between all-cause mortality and AST in the highest decile and DRR in tertile 3 compared with the respective reference categories, both in participants with hepatic steatosis, lost significance. The association between cancer mortality and low ALT in participants without hepatic steatosis compared with the reference deciles was also no longer significant. However, participants without hepatic steatosis in ALT deciles 1–3 had significantly higher heart disease mortality after additionally adjusting for frailty compared with the reference deciles (HR = 1.30, 95% CI = 1.08–1.57). In addition, AST in the highest decile in participants without hepatic steatosis was significantly associated with cancer mortality compared with the reference deciles (HR = 1.78, 95% CI = 1.07–2.96).

## 4. Discussion

In this nationally representative study, we observed generally higher all-cause mortality with low AST and ALT concentrations compared with intermediate AST and ALT concentrations, while associations with high transaminase concentrations often missed significance. In addition, high GGT concentration and high DRR were related to higher all-cause mortality. Interestingly, there seemed to be no difference in the association of liver enzymes or DRR with mortality between participants with and without NAFLD.

Compared with intermediate concentrations, low transaminases seem to be more likely to be related to mortality than high transaminases, which is already known from the literature [3,4,5,6]. The groups with the highest decile generally had smaller sample sizes, fewer deaths, and larger confidence intervals than the groups with the lowest deciles. Only the association between AST decile 10 in participants with NAFLD and all-cause mortality was significant. In the study by Gallo et al., having excluded patients with a history of chronic liver disease, high AST was significantly associated with all-cause mortality, whereas ALT missed significance [6]. The relatively small numbers of participants and cause-specific deaths may also account for the few associations with cause-specific mortality, which have also been inconsistently reported in the literature [2,3,5,6].

The association between low transaminases and higher mortality is already known from numerous studies and is confirmed by our results; however, none of these studies excluded or subdivided by sonographically diagnosed NAFLD [2,3,5,6,11,12,39,40,41]. Many underlying causes have been considered, especially regarding low ALT: liver aging [6,11,39], malnutrition [11,12], sarcopenia [5,12], frailty [12,39], and low BMI [5,39]. Previous studies have shown an association between low ALT and frailty [12,39,40], as well as advanced age [39], low BMI [5,11,39], sarcopenia [5,12], and pyridoxine deficiency [12]. Low BMI is associated with poorer outcome, especially in the elderly, whose estimated BMI for lowest risk of all-cause mortality is higher than in younger patients [42]. As a possible explanation for the relation between ALT and BMI, le Couteur et al., who did not examine whether participants had NAFLD or not, considered the absence of NAFLD in nonobese patients and therefore low transaminases [39]. However, in our study, mortality was higher with low transaminases, even after adjusting for BMI, and in both participants with and without NAFLD, suggesting that this, at least, is not the only cause. Many of the mechanisms above are also risk factors for frailty: advanced age, malnutrition, micronutrient deficits e.g., vitamin B6, and lack of exercise [43]. Physical inactivity, low protein intake, and micronutrient deficiency lead to loss of muscle mass, strength, and function, which are characteristics of sarcopenia. Therefore, sarcopenia is closely related to frailty [44]. In the study of Le Couteur et al., the association between low ALT and mortality disappeared after adjusting for frailty and age, suggesting the association might be mediated by these factors [39]. In contrast, our results remained significant. To note, in the cohort of Le Couteur et al., the prevalence of frailty was higher because they only included elderly patients [39]. Since our data is cross-sectional, we cannot exclude the possibility that some participants were already at risk for frailty but did not yet meet diagnostic criteria. The current literature is inconsistent not only in terms of ALT and frailty but also in terms of sarcopenia. Le Couteur et al. could not find an association between lean body mass and ALT. They hypothesized that hepatic mechanisms were responsible for the association between low ALT and higher mortality, rather than sarcopenia, because ALT correlated with AST and GGT [39]. On the other hand, Ruhl et al. was able to show an association between ALT and appendicular lean mass [5]. A further frequently considered mechanism is malnutrition [11,12]. As part of malnutrition, pyridoxine deficiency is associated with low ALT [12] and high DRR [45].

Nevertheless, it seems that none of the above discussed mechanisms can entirely explain the association between low ALT and higher mortality. Although Vespasiani et al. showed that low ALT was significantly associated with frailty, disability, sarcopenia, and pyridoxine deficiency, the association of low ALT and higher mortality risk remained significant after adjusting for all four confounders simultaneously. As noted by Vespasiani et al., other yet unrecognized biological factors may be responsible [12]. Possibly, low ALT reflects hepatic aging rather than overall aging, as proposed by Elinav et al. [11]. Le Couteur et al. supported this hypothesis because ALT correlated with AST and GGT, likely reflecting hepatic origin [39]. Similarly, Gallo et al. concluded that low transaminases seem to be not only markers of sarcopenia, frailty, or disability, but also of yet unrecognized mechanisms, e.g., hepatic aging [6].

Consistent with previous studies, high GGT [2,3,4] and DRR [3] were significantly associated with all-cause mortality in participants both with and without hepatic steatosis; however, those studies did not distinguish between patients with and without NAFLD. There is one study by Haring et al. that demonstrated a stronger association between GGT and all-cause mortality in men with a hyperechogenic ultrasound pattern than in men without such pattern but not in women. However, they adjusted for, among other things, comorbidities, and the number of deaths and participants with hepatic steatosis was small [13]. In contrast, without adjusting for comorbidities, our multivariate-adjusted results showed similar HR and 95% CI for participants with and without hepatic steatosis. It is known that GGT is elevated not only in liver diseases [1] and thus could be elevated by non-hepatic comorbidities. GGT was associated with oxidative stress [9] and inflammation [10]. The lack of specificity of GGT for liver disease might be the reason for missing differences in mortality between participants with and without hepatic steatosis. DRR can be used as a marker for progressive liver disease. A ratio >1 is considered indicative of moderate to severe fibrosis or cirrhosis [15,46]. However, DRR is affected not only by hepatocellular damage but also by extrahepatic disorders [45,47], as it is defined as AST over ALT [8], and AST in particular is lacking specificity for liver injury [1]. Supporting this, DRR tertile 3 in participants with hepatic steatosis was not more strongly associated with mortality than in participants without hepatic steatosis.

Regarding hepatic steatosis, NAFLD seems to be inconsistently associated with mortality. In a meta-analysis by Musso et al., NAFLD was significantly associated with overall mortality, mainly by cardiovascular and liver disease mortality [19]. Using NHANES III data with shorter follow-up, Unalp-Arida et al. were able to show a significant association between sonographically severe NAFLD and liver disease mortality but not with all-cause or cardiovascular disease mortality. They discussed genetic heterogeneity resulting in different metabolic subtypes as underlying causes of NAFLD as a possible reason [4]. In the study of Younossi et al., also using NHANES III data, participants with NAFLD and MetS had a higher risk of all-cause and cardiovascular disease mortality, whereas patients with NAFLD but without MetS did not [20]. It is discussed whether elevated liver enzymes are associated with mortality due to MetS, which causes NAFLD and is also associated with many non-liver diseases [17,18]. However, because our results suggest that there is no difference in the association of liver enzymes and mortality between participants with and without NAFLD, other non-hepatic diseases appear to be responsible. Yuwaki et al. reported that elevated liver enzymes were still associated with all-cause and non-liver disease mortality even after excluding participants with viral hepatitis [18]. Yuwaki et al. and Turati et al. considered the following as possible causes of the association between elevated liver enzymes and non-liver disease mortality: liver disease as a possibly aggravating comorbidity, shared common risk factors of liver and non-liver diseases, and other organs releasing liver enzymes liver-independently [17,18]. Known non-hepatic causes for elevated transaminases are, e.g., cardiac muscle damage, hemolysis, skeletal muscle damage, rhabdomyolysis, thyroid disease, and adrenal insufficiency [1]. Another reason could be the poor correlation between liver enzymes, especially ALT, and histological severity of NAFLD [21,22,23,24]. Despite normal enzyme levels, progressive disease or fibrosis cannot be ruled out [21,22,23,24], and progressive diseases, especially fibrosis, are critical prognostic factors [48]. It is possible that participants with progressive diseases or even fibrosis were included in the reference groups.

A very recently published study proposed a new nomenclature and diagnostic criteria for fatty liver disease. Rinella et al. suggested replacing NAFLD with the term metabolic dysfunction-associated steatotic liver disease (MASLD) and defined it as the presence of hepatic steatosis and at least one cardiometabolic criterion in the absence of other possible causes [49]. Furthermore, they introduced the new term MetALD, which refers to patients with MASLD and an average daily alcohol consumption of 30–60 g in men and 20–50 g in women [49]. To address the most recent proposed changes in nomenclature, we considered how they aligned with our study. Because we used a low threshold for alcohol consumption as an exclusion criterion, none of our participants with NAFLD met the criteria for MetALD. Among the 11,385 participants in the analysis sample for AST, ALT, and DRR, 2643 were diagnosed with NAFLD. Using available data, we examined how many of the 2643 NAFLD participants also had at least one cardiometabolic criterion. The criteria used were BMI ≥25 kg/m^2^, physician-diagnosed hypertension, HbA1c ≥5.7%, physician-diagnosed diabetes, serum triglycerides ≥150 mg/dL, and serum HDL ≤40 mg/dL in men and ≤50 mg/dL in women. A total of 2469 (93.4% of 2643 NAFLD participants) met criteria for MASLD, resulting in an absolute difference of 174 participants in whom we diagnosed NAFLD but who did not meet criteria for MASLD.

Because we were not able to apply all the proposed cardiometabolic criteria and because Rinella et al. also suggested using the term possible MASLD for participants with high suspicion of MASLD but without cardiometabolic criteria until additional testing had taken place [49], we believe that the NAFLD participants in our study are most consistent with patients diagnosed with MASLD.

## Strengths and Limitations

There were several limitations in this study. First, because NHANES III is a cross-sectional study, serum concentrations of liver enzymes were obtained only at baseline, and hepatic steatosis was assessed once, participants may have been misclassified. Furthermore, the gold standard to assess NAFLD is liver biopsy. However, it is not possible to conduct liver biopsies on the general population. Ultrasound is a non-invasive and cheap alternative [15]. Third, we were unable to examine liver disease mortality due to the restrictions of the NHANES III mortality dataset. Lastly, using death certificate diagnoses for assigning cause of death is prone to misclassification. Therefore, analyses with cause-specific mortality might be less reliable than those with all-cause mortality.

Despite those limitations, there were also several strengths. With up to 31 years of follow-up, the number of deaths was high, and we were able to study long-term effects of liver enzymes on mortality. Another strength of this study is that we additionally adjusted for frailty, as this is a commonly discussed mechanism for the association between low ALT and high mortality. Finally, using sample weights, the results can be generalized to the U.S. population because of its design as a large, population-based sample without the bias of clinical trials where inclusion criteria are applied to select participants.

## 5. Conclusions

In conclusion, there seems to be no difference in the association of liver enzymes or DRR and mortality between participants with and without NAFLD. A poor correlation between liver enzymes and severity of NAFLD could be a reason. Hence, our results suggest that high liver enzyme levels are associated with mortality not only due to hepatocellular damage but also because of systemic disorders. Furthermore, these findings support the hypothesis that low transaminases are likely to be associated with higher mortality due to liver senescence, which may affect participants with and without NAFLD.

## Figures and Tables

**Table 1:** Baseline characteristics of the study cohorts by hepatic steatosis of the National Health and Nutrition Examination Survey III (1988–1994), United States.

	AST, ALT, DRR Cohort		GGT Cohort	
	without Hepatic Steatosis	with Hepatic Steatosis	without Hepatic Steatosis	with Hepatic Steatosis
	*n* = 8742	*n* = 2643	*n* = 6804	*n* = 2075
Age (years)	39 (29–52)	46 (35–60)	39 (29–52)	46 (35–58)
Females	53	45.8	52.6	45.4
Race/Ethnicity				
Non-Hispanic white	76.3	76.2	75.6	74.6
Non-Hispanic black	10.8	8.3	11.2	9
Mexican American	5	7.5	4.6	7.3
Other	7.9	7.9	8.6	9
Education				
<12 years	21	28.8	20.8	27.6
12 years	34.2	36.8	33.4	37.3
>12 years	44.7	34.4	45.9	35.1
No alcohol consumption	42	49.1	41.9	49.4
Smoking				
Never	47.1	42.9	48	42.8
Former	23.1	34	22.6	34
Current	29.9	23.1	29.4	23.3
LTPA				
No LTPA	12.3	16.6	12.6	16.8
Irregular LTPA	39.2	42.2	38.8	41
Regular LTPA	48.5	41.2	48.6	42.1
BMI (kg/m^2^)	24.9 (22.3–28.1)	29.6 (26.3–33.8)	25 (22.3–28.1)	29.6 (26.2–33.9)
Diabetes	4.4	15.5	4.3	15.3
Hypertension	18.8	35.6	18.6	34.7
Frailty	1.4	2.1	1.3	1.9
Serum HDL < 35 mg/dL	9.4	23.9	9.4	24.9
Serum cholesterol				
<200 mg/dL	52.5	39	53.1	39.8
200–239 mg/dL	30.5	35.2	30.4	34.8
≥240 mg/dL	16.9	25.9	16.6	25.4
Serum triglycerides				
<250 mg/dL	93.2	74.9	93.3	75.8
250–500 mg/dL	6	21	5.9	20.1
>500 mg/dL	0.8	4.2	0.8	4.1
AST (U/L)	18 (16–22)	21 (18–27)	18 (16–22)	21 (18–27)
ALT (U/L)	13 (10–19)	20 (14–29)	13 (10–19)	20 (14–29)
GGT (U/L)	18 (13–27)	29 (20–43)	18 (13–27)	29 (20–43)
DRR	1.4 (1.1–1.7)	1.1 (0.9–1.4)	1.4 (1.1–1.7)	1.1 (0.8–1.4)
Serum albumin (g/dL)	4.2 (4–4.4)	4.2 (4–4.4)	4.2 (4–4.4)	4.2 (3.9–4.4)
Total bilirubin (mg/dL)	0.5 (0.4–0.7)	0.5 (0.4–0.7)	0.5 (0.4–0.7)	0.6 (0.4–0.7)
Platelets (G/L)	264 (226–307.5)	265.5 (224.5–315)	262 (223–305)	261 (221.5–311)
CRP >0.3 mg/dL	21.8	36.8	23.9	39.6
eGFR < 60 mL/min/1.73 m^2^	8.6	13.1	8.8	12.5

Abbreviations: AST = aspartate aminotransferase, ALT = alanine aminotransferase, DRR = De Ritis ratio, GGT = gamma glutamyltransferase, LTPA = leisure-time physical activity, BMI = body mass index, HDL = high-density lipoprotein, CRP = C-reactive protein, eGFR = estimated glomerular filtration rate, IQR = interquartile range. Continuous variables presented as median (IQR); categorical variables presented as percentage. Number *n* is unweighted.

**Table 2:** Cumulative probability of mortality (unadjusted) over a 31-year period and age-adjusted hazard ratios for mortality by hepatic steatosis and liver enzyme decile or DRR tertile (*n* = 11,385 in analysis samples for AST, ALT, and DRR; *n* = 8879 in analysis sample for GGT) of the National Health and Nutrition Examination Survey III (1988–1994), United States.

	without Hepatic Steatosis				with Hepatic Steatosis			
Mortality Outcome and Liver Enzyme Decile or DRR Tertile ^a^	No. of Deaths ^b^	Unadjusted Cumulative Mortality ^c^	Age-Adjusted HR ^d^	95% CI	No. of Deaths ^b^	Unadjusted Cumulative Mortality ^c^	Age-Adjusted HR ^d^	95% CI
AST								
All-cause								
Deciles 1–3	925	30.4	1.54	1.37–1.73	290	42.8	1.21	1.01–1.46
Deciles 4–9 (ref.)	1675	30.2	1.00	-	695	44.7	1.00	-
Decile 10	208	35.6	1.2	0.94–1.54	207	45.9	1.3	1.02–1.66
Heart disease								
Deciles 1–3	247	7.4	1.44	1.15–1.81	85	13.7	1.19	0.87–1.63
Deciles 4–9 (ref.)	480	10.5	1.00	-	207	14.4	1.00	-
Decile 10	58	10.7	1.15	0.79–1.68	40	12	0.87	0.55–1.4
Cancer								
Deciles 1–3	275	12.2	2.2	1.7–2.85	55	11.9	1.15	0.82–1.6
Deciles 4–9 (ref.)	375	7.4	1.00	-	166	12.2	1.00	-
Decile 10	43	14	1.54	0.93–2.56	39	13	1.12	0.63–1.98
ALT								
All-cause								
Deciles 1–3	1000	34.7	1.33	1.19–1.49	227	53	1.42	1.17–1.73
Deciles 4–9 (ref.)	1680	29	1.00	-	775	46.4	1.00	-
Decile 10	128	23.4	1.24	0.85–1.81	190	34	1.18	0.93–1.5
Heart disease								
Deciles 1–3	283	9.6	1.26	1.01–1.57	73	21	1.63	1.16–2.29
Deciles 4–9 (ref.)	475	9.6	1.00	-	219	14.5	1.00	-
Decile 10	27	4.8	0.94	0.48–1.85	40	8.3	1.03	0.64–1.68
Cancer								
Deciles 1–3	274	12.4	1.64	1.32–2.03	48	15.3	1.39	0.84–2.29
Deciles 4–9 (ref.)	386	8.2	1.00	-	171	13.3	1.00	-
Decile 10	33	7.6	1.39	0.8–2.43	41	7.5	0.83	0.43–1.59
GGT								
All-cause								
Deciles 1–8 (ref.)	1649	25.6	1.00	-	578	37.5	1.00	-
Deciles 9–10	427	39.1	1.47	1.21–1.79	306	45.6	1.42	1.17–1.72
Heart disease								
Deciles 1–8 (ref.)	441	6.9	1.00	-	166	12	1.00	-
Deciles 9–10	123	11.3	1.46	0.91–2.36	81	12	1.17	0.78–1.76
Cancer								
Deciles 1–8 (ref.)	420	7.9	1.00	-	136	9.6	1.00	-
Deciles 9–10	95	11.6	1.35	0.94–1.93	63	13	1.35	0.86–2.14
DRR								
All-cause								
Tertile 1 (ref.)	699	25.5	1.00	-	520	38.2	1.00	-
Tertile 2	1019	30.3	0.95	0.81–1.11	390	53.6	1.12	0.94–1.33
Tertile 3	1090	35.3	1.07	0.94–1.21	282	56.4	1.11	0.92–1.34
Heart disease								
Tertile 1 (ref.)	185	8.1	1.00	-	144	9.9	1.00	-
Tertile 2	279	8.5	0.96	0.69–1.33	107	20	1.07	0.79–1.44
Tertile 3	321	11.2	1.19	0.88–1.61	81	21.3	1.15	0.8–1.64
Cancer								
Tertile 1 (ref.)	176	7.1	1.00	-	119	10.1	1.00	-
Tertile 2	252	10.3	0.99	0.74–1.34	84	15.2	1.29	0.87–1.9
Tertile 3	265	10.7	1.06	0.84–1.34	57	16.6	1.15	0.69–1.91

Abbreviations: DRR = De Ritis ratio, AST = aspartate aminotransferase, ALT = alanine aminotransferase, GGT = gamma glutamyltransferase, HR = hazard ratio, CI = confidence interval. ^a^ AST cut-points were 18 and 34 U/L for men and 16 and 27 U/L for women. ALT cut-points were 13 and 36 U/L for men and 9 and 24 U/L for women. GGT cut-points were 46 U/L for men and 31 U/L for women. DRR cut-points were 1.2 and 1.64. ^b^ Number of deaths is unweighted. ^c^ Estimated using Kaplan-Meier analysis. ^d^ Estimated using Cox proportional hazards regression analysis with age as a continuous variable.

## Data Availability

Data from the National Health and Nutrition Examination Survey III are freely available from the U.S. Centers for Disease Control and Prevention (https://wwwn.cdc.gov/nchs/nhanes/nhanes3/datafiles.aspx (accessed on 17 June 2022)).

## Supplementary Materials

The following supporting information can be downloaded at https://www.mdpi.com/article/10.3390/nu15133063/s1: Table S1: Baseline characteristics by hepatic steatosis and AST decile; Table S2: Baseline characteristics by hepatic steatosis and ALT decile; Table S3: Baseline characteristics by hepatic steatosis and GGT decile; Table S4: Baseline characteristics by hepatic steatosis and DRR tertile; Figure S1: Multivariate-adjusted hazard ratios with 95% confidence intervals for (a) all-cause, (b) heart disease, and (c) cancer mortality by hepatic steatosis and liver enzyme decile or DRR tertile after excluding participants taking certain medications that may cause hepatic steatosis (*n* = 11,375 in analysis samples for AST, ALT, and DRR; *n* = 8871 in analysis sample for GGT).
