# Supplementary material for: The Association between Liver Enzymes and Mortality Stratified by Non-Alcoholic Fatty Liver Disease: An Analysis of NHANES III
Source: Nutrients. 2023 Jul 7;15(13):3063. doi: 10.3390/nu15133063 (PMC10346959; doi:10.3390/nu15133063)
Supplement: Supplementary file 1 [file nutrients-15-03063-s001.zip › nutrients-2467130-supplementary.pdf]

Supplementary Table S1. Baseline characteristics by hepatic steatosis and AST decile of the National Health and Nutrition Examination Survey III (1988-1994), United States.

	without hepatic steatosis			with hepatic steatosis		
	AST decile[a]			AST decile[a]		
	1-3 (N=2863)	4-9 (N=5330)	10 (N=549)	1-3 (N=580)	4-9 (N=1589)	10 (N=474)
Age (years)	37 (29-48)	39 (29-53)	40 (32-54)	46 (33-60)	47 (36-60)	46 (33-57)
Females	51.9	53.3	56.9	49.9	41.9	55.3
Race/Ethnicity						
Non-Hispanic white	78.1	75.7	70.9	77.6	77.3	70
Non-Hispanic black	10.5	10.8	12.6	10.3	7.8	7.4
Mexican-American	4.5	5.1	7.2	5.4	6.9	12.9
Other	6.9	8.4	9.3	6.6	8	9.7
Education						
<12 years	22.8	19.8	24.1	32.2	27.8	28.1
12 years	35.3	33.9	31.6	38.7	34.7	42.1
>12 years	41.8	46.4	44.3	29.1	37.5	29.8
No alcohol consumption	43	41	47.4	49.6	48.7	50
Smoking						
Never	39.5	50.8	52.9	37.3	43.8	47.3
Former	22.9	23.4	20.3	32.6	35.1	31.7
Current	37.6	25.8	26.8	30.2	21.1	21
LTPA						
No LTPA	14	11.4	12.2	18.2	16.6	14.8
Irregular LTPA	43.1	37.6	32.5	43.7	41.2	44
Regular LTPA	43	51	55.4	38.1	42.3	41.2
BMI (kg/m^2)	24.8 (22.1-28)	25 (22.4-28)	25.6 (22.8-29.1)	28.7 (25.4-33.3)	29.6 (26.4-33.7)	30.6 (27.2-35.8)
Diabetes	5.4	3.7	5.1	17.6	14	18.2
Hypertension	18.1	19	21.2	35.5	34.5	40.1
Frailty	1.6	1.4	1.2	2.5	2.2	1.3
Serum HDL <35 mg/dL	12.1	7.8	11.1	20.4	24.8	25.5
Serum cholesterol						
<200 mg/dL	57.4	50	49.8	43.1	38.3	35.8
200-239 mg/dL	29.2	31.4	29.4	36.2	36.3	29.7
≥240 mg/dL	13.4	18.6	20.8	20.7	25.4	34.5
Serum triglycerides						
<250 mg/dL	94.3	92.8	89.7	82.3	74.5	66.1
250-500 mg/dL	5.1	6.3	8.9	15.7	21.2	27
>500 mg/dL	0.5	0.8	1.4	2	4.3	6.8
AST (U/L)	15 (14-16)	20 (18-23)	36 (30-43)	15 (14-16)	22 (19-25)	38 (34-49)
ALT (U/L)	11 (8-14)	15 (11-20)	31 (22-43)	12 (9-17)	20 (15-27)	42 (28-62)
GGT (U/L)	16 (13-22)	19 (14-29)	36 (19-67)	22 (15-29)	29 (21-41)	53 (30-88)
DRR	1.4 (1.1-1.7)	1.4 (1.1-1.7)	1.2 (0.9-1.6)	1.2 (0.9-1.5)	1.1 (0.9-1.4)	1 (0.7-1.4)
Serum albumin (g/dL)	4.2 (4-4.4)	4.2 (4-4.5)	4.2 (4-4.5)	4.1 (3.9-4.4)	4.2 (4-4.4)	4.2 (4-4.4)
Total bilirubin (mg/dL)	0.5 (0.4-0.7)	0.6 (0.4-0.7)	0.6 (0.4-0.7)	0.5 (0.4-0.7)	0.6 (0.4-0.7)	0.6 (0.4-0.7)
Platelets (G/L)	268 (226-312.5)	263 (226-304)	255 (226.5-319)	273.5 (229-321)	267.5 (226.5-315)	257 (212.5-303)
CRP >0.3 mg/dL	24	19.9	28.5	40	33.9	44
eGFR <60 ml/min/1.73m^2	7	9.3	10.7	12.6	12.9	14.5

Abbreviations: AST = aspartate aminotransferase, LTPA = leisure-time physical activity, BMI = body mass index, HDL = high-density lipoprotein, ALT = alanine aminotransferase, GGT = gamma glutamyltransferase, DRR = De Ritis ratio, CRP = C-reactive protein, eGFR = estimated glomerular filtration rate, IQR = interquartile range.

Continuous variables presented as median (IQR); categorical variables presented as percentage. Number N is unweighted.

[a]AST cut-points were 18 and 34 U/L for men and 16 and 27 U/L for women.

Supplementary Table S2. Baseline characteristics by hepatic steatosis and ALT decile of the National Health and Nutrition Examination Survey III (1988-1994), United States.

	**without hepatic steatosis**			**with hepatic steatosis**		
	**ALT decile[a]**			**ALT decile[a]**		
	**1-3 (N=2627)**	**4-9 (N=5563)**	**10 (N=552)**	**1-3 (N=380)**	**4-9 (N=1682)**	**10 (N=581)**
Age (years)	39 (28-54)	39 (30-52)	35 (27-46)	50 (32-65)	48 (38-60)	39 (33-52)
Females	56.6	51	57.7	55.1	41	54.5
Race/Ethnicity						
Non-Hispanic white	75.5	77.1	71.2	77.4	77.4	72.2
Non-Hispanic black	14.3	9.4	9.4	16.6	8	4.4
Mexican-American	3.5	5.3	9.6	3.7	6.6	12.4
Other	6.7	8.3	9.8	2.3	8	11
Education						
<12 years	23.2	19.9	22.7	38.4	29.1	22.5
12 years	34.6	34.2	32.5	38.8	35.5	39.2
>12 years	42.2	45.8	44.8	22.7	35.4	38.3
No alcohol consumption	43.3	41.2	44.6	53.1	47	53.1
Smoking						
Never	42.3	48.7	52.8	36.4	41.6	50.4
Former	22	24	16.9	29.8	35.6	31.7
Current	35.6	27.3	30.3	33.8	22.8	17.9
LTPA						
No LTPA	14.7	11.3	12	18.2	16.1	17.3
Irregular LTPA	38.8	38.9	45.1	43.9	41.7	42.5
Regular LTPA	46.5	49.9	42.9	37.9	42.2	40.2
BMI (kg/m^2)	23.8 (21.5-26.8)	25.3 (22.7-28.4)	26 (23.6-29.5)	25.9 (22-30.3)	29.5 (26.4-33.7)	31.2 (27.8-36.2)
Diabetes	3.6	4.4	8.1	12.2	14.4	20.4
Hypertension	17.9	19.2	19	30.8	36.4	36.1
Frailty	2.1	1.3	0.8	1.8	1.6	3.9
Serum HDL <35 mg/dL	8.6	9.6	11.5	12.2	24.4	29.2
Serum cholesterol						
<200 mg/dL	58	50.6	46.1	47.1	38	37.3
200-239 mg/dL	28	31.3	34.9	32.9	36.4	33
≥240 mg/dL	14	18.1	18.9	20	25.6	29.8
Serum triglycerides						
<250 mg/dL	95.9	92.4	88.1	88.1	75.3	66.1
250-500 mg/dL	3.9	6.6	10.9	10.1	20.4	28.8
>500 mg/dL	0.2	1	1	1.8	4.3	5.1
AST (U/L)	16 (14-18)	19 (17-22)	29 (25-38)	16 (14-18)	20 (18-24)	32 (26-43)
ALT (U/L)	9 (7-10)	15 (13-20)	38 (30-47)	9 (7-10)	19 (15-23)	40 (30-54)
GGT (U/L)	15 (12-20)	20 (14-29)	36 (22-68)	17 (12-22)	27 (20-38)	43 (30-69)
DRR	1.9 (1.6-2.2)	1.2 (1-1.5)	0.8 (0.6-1)	1.9 (1.6-2.2)	1.1 (0.9-1.4)	0.8 (0.7-1)
Serum albumin (g/dL)	4.2 (4-4.4)	4.2 (4-4.4)	4.2 (3.9-4.4)	4.1 (3.9-4.4)	4.2 (4-4.4)	4.2 (4-4.4)
Total bilirubin (mg/dL)	0.5 (0.4-0.7)	0.6 (0.4-0.7)	0.5 (0.4-0.7)	0.4 (0.4-0.6)	0.6 (0.4-0.7)	0.6 (0.4-0.7)
Platelets (G/L)	265 (225.5-307.5)	263.5 (225.5-306)	262 (231.5-332)	270 (231-340)	265.5 (224.5-313)	263.5 (221-316.5)
CRP >0.3 mg/dL	23.3	20.6	27.2	39.1	34.5	42.4
eGFR <60 ml/min/1.73m^2	11	7.9	4.2	14	13.9	10.3

Abbreviations: ALT = alanine aminotransferase, LTPA = leisure-time physical activity, BMI = body mass index, HDL = high-density lipoprotein, AST = aspartate aminotransferase, GGT = gamma glutamyltransferase, DRR = De Ritis ratio, CRP = C-reactive protein, eGFR = estimated glomerular filtration rate, IQR = interquartile range.
Continuous variables presented as median (IQR); categorical variables presented as percentage. Number N is unweighted.
[a]ALT cut-points were 13 and 36 U/L for men and 9 and 24 U/L for women.

Supplementary Table S3. Baseline characteristics by hepatic steatosis and GGT decile of the National Health and Nutrition Examination Survey III (1988-1994), United States.

	**without hepatic steatosis**		**with hepatic steatosis**	
	**GGT decile[a]**		**GGT decile[a]**	
	**1-8 (N=5707)**	**9-10 (N=1097)**	**1-8 (N=1398)**	**9-10 (N=677)**
Age (years)	38 (29-51)	42 (33-56)	45 (33-59)	48 (37-58)
Females	52.5	53.1	42.6	52.2
Race/Ethnicity				
Non-Hispanic white	77.3	63.1	78	66.4
Non-Hispanic black	9.8	21.6	8.1	11.4
Mexican-American	4.4	5.9	6.4	9.5
Other	8.5	9.5	7.5	12.6
Education				
<12 years	19.9	27.1	25.1	33.7
12 years	33.1	35.5	39.2	32.9
>12 years	47	37.4	35.7	33.4
No alcohol consumption	42.1	40.1	49.5	49
Smoking				
Never	48.8	42.5	42.1	44.4
Former	22.4	23.8	33.6	34.7
Current	28.8	33.7	24.2	20.9
LTPA				
No LTPA	11.8	18.4	15.3	20.8
Irregular LTPA	38.9	37.6	41	41.2
Regular LTPA	49.2	44	43.8	38.1
BMI (kg/m^2)	24.8 (22.2-27.9)	26.5 (23.7-30.5)	29.1 (25.8-33.3)	30.7 (27.2-35.7)
Diabetes	3.5	10	12.5	22.1
Hypertension	17	30.4	33.6	37.4
Frailty	1	3.8	1.7	2.5
Serum HDL <35 mg/dL	9.4	9.7	25.3	24
Serum cholesterol				
<200 mg/dL	54.9	39.9	42.6	33
200-239 mg/dL	29.6	36.1	36.5	30.6
≥240 mg/dL	15.5	24	20.9	36.4
Serum triglycerides				
<250 mg/dL	94.3	86.1	79.2	67.2
250-500 mg/dL	5	12.4	17.7	26.1
>500 mg/dL	0.6	1.5	3.1	6.6
AST (U/L)	18 (16-21)	21 (18-28)	20 (17-25)	26 (20-37)
ALT (U/L)	13 (10-18)	19 (14-28)	18 (13-26)	28 (19-42)
GGT (U/L)	17 (13-23)	54 (42-77)	22 (18-29)	59 (46-86)
DRR	1.4 (1.1-1.7)	1.2 (0.9-1.4)	1.1 (0.9-1.5)	1 (0.7-1.2)
Serum albumin (g/dL)	4.2 (4-4.4)	4.1 (3.9-4.3)	4.2 (4-4.4)	4.1 (3.9-4.4)
Total bilirubin (mg/dL)	0.5 (0.4-0.7)	0.5 (0.4-0.7)	0.6 (0.4-0.7)	0.5 (0.4-0.7)
Platelets (G/L)	261.5 (222-302)	268.5 (228.5-324.5)	258 (222.5-307.5)	266 (219.5-316.5)
CRP >0.3 mg/dL	21.6	40.8	33.6	54
eGFR <60 ml/min/1.73m^2	8.4	11.5	11.7	14.4

Abbreviations: GGT = gamma glutamyltransferase, LTPA = leisure-time physical activity, BMI = body mass index, HDL = high-density lipoprotein, AST = aspartate aminotransferase, ALT = alanine aminotransferase, DRR = De Ritis ratio, CRP = C-reactive protein, eGFR = estimated glomerular filtration rate, IQR = interquartile range.

Continuous variables presented as median (IQR); categorical variables presented as percentage. Number N is unweighted.

[a]GGT cut-points were 46 U/L for men and 31 U/L for women.

Supplementary Table S4. Baseline characteristics by hepatic steatosis and DRR tertile of the National Health and Nutrition Examination Survey III (1988-1994), United States.

	without hepatic steatosis			with hepatic steatosis		
	DRR tertile[a]			DRR tertile[a]		
	Tertile 1 (N=2786)	Tertile 2 (N=3210)	Tertile 3 (N=2746)	Tertile 1 (N=1444)	Tertile 2 (N=720)	Tertile 3 (N=479)
Age (years)	37 (29-47)	40 (30-52)	40 (29-57)	44 (34-54)	53 (40-64)	54 (36-66)
Females	36.1	57.2	67.2	36.8	57	63.5
Race/Ethnicity						
Non-Hispanic white	76.1	77.9	74.5	75.7	78.6	74.8
Non-Hispanic black	7.6	10.4	15	5	10.8	17
Mexican-American	6.6	4.9	3.3	8.7	6.6	4.5
Other	9.7	6.8	7.2	10.6	4	3.7
Education						
<12 years	19.5	21.7	22	26	32.7	33.5
12 years	33.7	33.8	35.5	35.8	38.8	37.5
>12 years	46.8	44.5	42.5	38.2	28.5	29
No alcohol consumption	40.1	42.9	43.1	47.4	48.7	56.4
Smoking						
Never	45.9	47.9	47.4	42.7	43.5	43
Former	22.8	24.8	21.2	35.6	32.8	29.5
Current	31.3	27.3	31.4	21.7	23.7	27.5
LTPA						
No LTPA	11	12.3	13.9	17.1	14.9	17.3
Irregular LTPA	42.5	37.8	37	42.9	43	38.3
Regular LTPA	46.5	49.9	49.1	40	42	44.5
BMI (kg/m^2)	26.1 (23.7-29.3)	24.9 (22.3-28)	23.6 (21.3-26.5)	30.6 (27.3-34.5)	28.4 (25.3-32.5)	26.8 (22.8-32.2)
Diabetes	6.4	3.5	3.2	17.2	13.4	11.7
Hypertension	18.6	18.8	19.1	34.1	40	34.8
Frailty	1	1.2	2.3	2.4	1.7	1.8
Serum HDL <35 mg/dL	13.9	8.5	5.5	29.6	15.5	14.8
Serum cholesterol						
<200 mg/dL	48.7	53.1	56.2	38.8	38.9	39.8
200-239 mg/dL	33	30.2	28.1	35.6	34.3	34.9
≥240 mg/dL	18.3	16.7	15.7	25.6	26.8	25.3
Serum triglycerides						
<250 mg/dL	90.4	93.4	96.2	71	79.2	83.1
250-500 mg/dL	8.6	5.8	3.4	24.1	17.6	13.7
>500 mg/dL	1	0.8	0.4	4.8	3.2	3.2
AST (U/L)	19 (16-24)	18 (16-21)	18 (15-21)	22 (19-29)	20 (17-24)	19 (17-26)
ALT (U/L)	20 (16-26)	13 (11-15)	9 (7-11)	26 (19-35)	14 (12-18)	10 (8-14)
GGT (U/L)	25 (17-35)	17 (13-25)	15 (12-20)	33 (24-49)	22 (17-33)	20 (14-32)
DRR	1 (0.8-1.1)	1.4 (1.3-1.5)	2 (1.8-2.3)	0.9 (0.7-1)	1.4 (1.3-1.5)	1.9 (1.8-2.2)
Serum albumin (g/dL)	4.2 (4-4.5)	4.2 (4-4.4)	4.2 (4-4.4)	4.2 (4-4.4)	4.2 (3.9-4.4)	4.1 (3.9-4.4)
Total bilirubin (mg/dL)	0.6 (0.4-0.7)	0.5 (0.4-0.7)	0.5 (0.4-0.7)	0.6 (0.4-0.7)	0.5 (0.4-0.7)	0.5 (0.4-0.7)
Platelets (G/L)	264.5 (227-306)	262.5 (224-310.5)	265.5 (227.5-306)	265.5 (223-315)	264.5 (226-306.5)	268.5 (227.5-340)
CRP >0.3 mg/dL	20.6	22	22.7	36.2	34.3	43
eGFR <60 ml/min/1.73m^2	4.8	8.4	13.2	9.2	20.1	17.4

Abbreviations: DRR = De Ritis ratio, LTPA = leisure-time physical activity, BMI = body mass index, HDL = high-density lipoprotein, AST = aspartate aminotransferase, ALT = alanine aminotransferase, GGT = gamma glutamyltransferase, CRP = C-reactive protein, eGFR = estimated glomerular filtration rate, IQR = interquartile range.

Continuous variables presented as median (IQR); categorical variables presented as percentage. Number N is unweighted.

[a]DRR cut-points were 1.2 and 1.64.

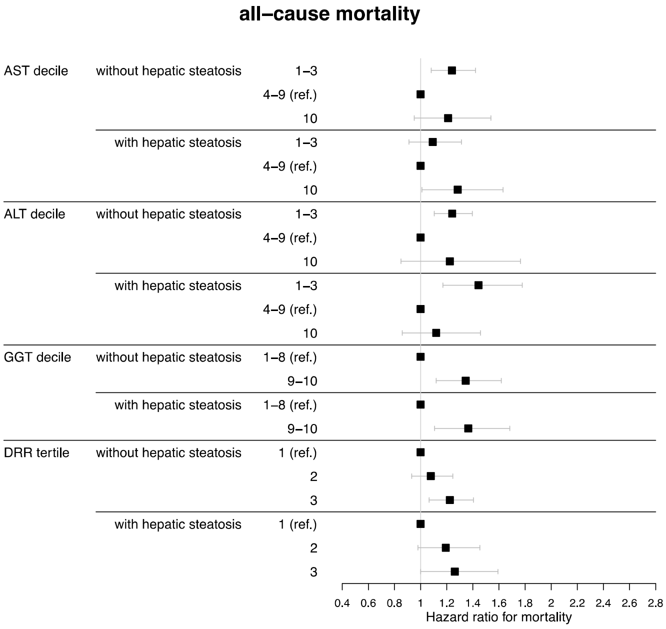

(**a**)

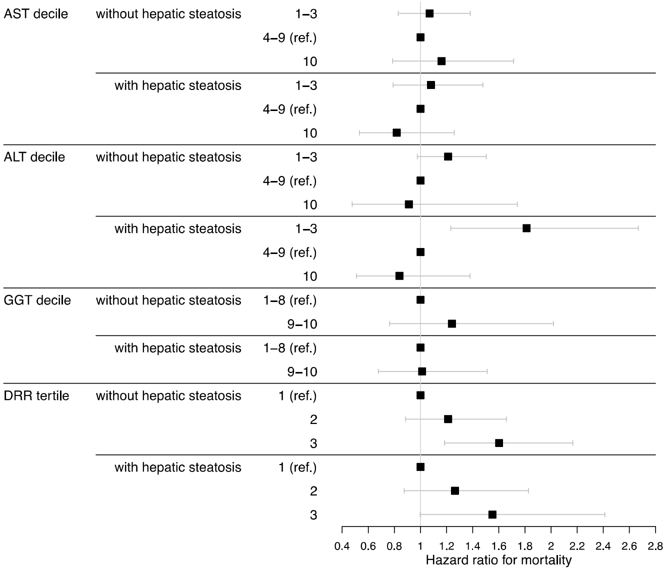

(**b**)

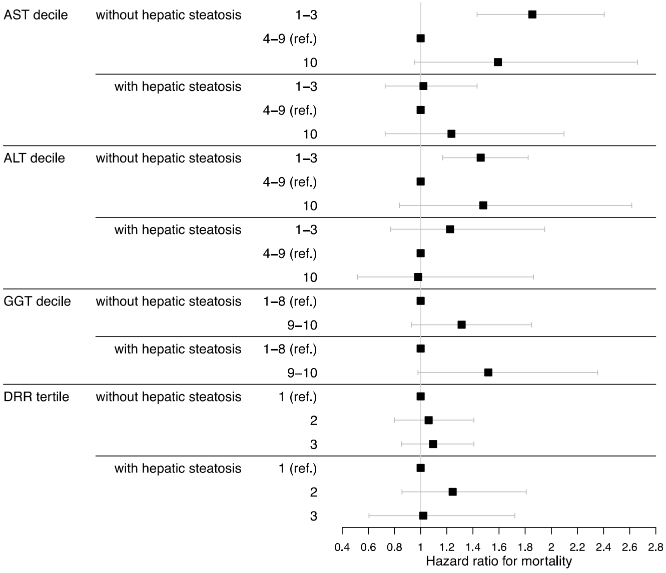

(**c**)

Supplementary Figure S1. Multivariate-adjusted hazard ratios with 95% confidence intervals for (**a**) all-cause, (**b**) heart disease, and (**c**) cancer mortality by hepatic steatosis and liver enzyme decile or DRR tertile after excluding participants taking certain medications that may cause hepatic steatosis (N=11,375 in analysis samples for AST, ALT, and DRR; N=8,871 in analysis sample for GGT) of the National Health and Nutrition Examination Survey III (1988-1994), United States. Abbreviations: AST = aspartate aminotransferase, ALT = alanine aminotransferase, GGT = gamma glutamyltransferase, DRR = De Ritis ratio. AST cut-points were 18 and 34 U/L for men and 16 and 27 U/L for women. ALT cut-points were 13 and 36 U/L for men and 9 and 24 U/L for women. GGT cut-points were 46 U/L for men and 31 U/L for women. DRR cut-points were 1.2 and 1.64. Hazard ratios were estimated using Cox proportional hazard regression analysis and adjusted for age, sex, race/ethnicity, education, alcohol consumption, cigarette smoking, leisure-time physical activity, body mass index, diabetes, hypertension, high-density lipoprotein, total cholesterol, and triglycerides.
